# HTLV-1 proviral load in adult T-cell leukemia/lymphoma (ATLL) patients from non-endemic regions of Argentina

**DOI:** 10.1186/1742-4690-11-S1-P34

**Published:** 2014-01-07

**Authors:** Andrea Mangano, María Saliba Pineda, Patricia Costantini, María Belén Bouzas, Cecilia Chidid, Griselda Di Stefano, Ana Inés Varela, Natalia Altamirano, Luciana Tadey, Lilia Mammana, Paula Aulicino, Liliana Olivares, Luisa Sen

**Affiliations:** 1 Laboratorio de Biología Celular y Retrovirus- CONICET, Hospital Garrahan, Ciudad Autónoma de Buenos Aires, Argentina; 2 División de Infectología, Hospital Angel H. Roffo, Ciudad Autónoma de Buenos Aires, Argentina; 3 Unidad de Virología, Hospital Muñiz, Ciudad Autónoma de Buenos Aires, Argentina; 4 Clínica Angelus, San Isidro, Provincia de Buenos Aires, Argentina; 5 División de Hematología, Hospital Angel H. Roffo, Ciudad Autónoma de Buenos Aires, Argentina; 6 Unidad Dermatología, Hospital Muñiz, Ciudad Autónoma de Buenos Aires, Argentina

## 

Few ATLL cases have been reported in the country, the majority of the acute leukemia subtype. HTLV-1 proviral load is considered a prognosis marker and it has been available in our setting since 2008. We present HTLV-1 proviral loads in 3 cases of ATLL received during the last 4 years. Two cases were of the acute subtype and one case of the lymphoma subtype. All were male with ages of 49, 34 and 54, respectively at diagnosis. Both acute cases were treated with CHOP chemotherapy. One was responder and one was chemo resistant. The latter after several treatments had disease progression and died 16 months after diagnosis. HTLV-1 proviral load was only measured at 10 months from diagnosis and was 5.3 log10/106 PBMC. The acute responder case had several measures of proviral load after clinical remission post-CHOP and during antiretroviral treatment plus alpha interferon. The proviral load was high but stable during almost four years (5.36 log10/106 PBMC). Afterwards, the patient was stopping treatment with interferon. Proviral load was 5.16 log10/106 PBMC and 6 log10/106 PBMC after one and 6 months, respectively. The lymphoma case was recently diagnosed (February 2013) had a proviral load of 3,76 log10/106 PBMC at that time and is currently untreated. The two acute leukemia cases showed similarly high proviral loads despite differences in survival time and response to treatment. Otherwise, in the lymphoma case proviral load was lower than in the acute leukemia cases. Further proviral load studies are needed to confirm this preliminary finding.

